# Self-Reported Physical Activity in Middle-Aged and Older Adults in Rural South Africa: Levels and Correlates

**DOI:** 10.3390/ijerph17176325

**Published:** 2020-08-31

**Authors:** Simone A. Tomaz, Justine I. Davies, Lisa K. Micklesfield, Alisha N. Wade, Kathleen Kahn, Stephen M. Tollman, Catherine E. Draper, Miles D. Witham

**Affiliations:** 1 Division of Exercise Science and Sports Medicine, Department of Human Biology, University of Cape Town, Cape Town 7700, South Africa; Catherine.Draper@wits.ac.za; 2 Faculty of Health Sciences and Sport, University of Stirling, Stirling, Scotland FK9 4LA, UK; 3 Institute for Applied Health Research, University of Birmingham, Birmingham B15 2TT, UK; j.davies.6@bham.ac.uk; 4 MRC/Wits Rural Public Health and Health Transitions Research Unit (Agincourt), School of Public Health, Faculty of Health Sciences, University of the Witwatersrand, Johannesburg 2000, South Africa; alisha.wade@wits.ac.za (A.N.W.); kathleen.kahn@wits.ac.za (K.K.); stephen.tollman@wits.ac.za (S.M.T.); 5 Department of Global Health, Stellenbosch University, Cape Town 7602, South Africa; 6 SAMRC/Wits Developmental Pathways for Health Research Unit, Faculty of Health Sciences, University of the Witwatersrand, Johannesburg 2000, South Africa; lisa.micklesfield@wits.ac.za; 7 AGE Research Group, NIHR Newcastle Biomedical Research Centre, Newcastle upon Tyne Hospitals NHS Foundation Trust and Faculty of Medical Sciences, Newcastle University, Newcastle upon Tyne NE1 7RU, UK

**Keywords:** functional capacity, aging, elderly

## Abstract

Little is known about physical activity (PA) levels and correlates in adults from rural settings in South Africa, where a rapid increase in the number of older people and marked disparities in wealth are evident, particularly between those living in rural and urban areas. This paper describes levels of self-reported PA in rural South African men and women and examines factors associated with meeting PA guidelines. Global Physical Activity Questionnaire (GPAQ) data from the Health and Aging in Africa: Longitudinal studies of INDEPTH communities (HAALSI) survey of 5059 adults aged over 40 years were assessed. Logistic regression analyses were used to assess socio-demographic, functional and cognitive capacity, and chronic disease measures associated with PA. In addition, 75.4% (*n* = 3421) of the participants with valid GPAQ data (*n* = 4538 of 5059) met the PA guidelines. Factors associated with not the meeting PA guidelines were being male, over the age of 80 years, being in a higher wealth category, obesity, and poorer functional capacity. These findings highlight worthwhile targets for future interventions to maintain or improve PA levels in this population and suggest that intervening earlier within this age range (from 40 years) may be crucial to prevent the ‘spiral of decline’ that characterizes the frailty syndrome.

## 1. Introduction

Low levels of physical activity (PA) are associated with multiple adverse health outcomes in adults, including cardiometabolic disease, functional decline, and premature death [1]. Although there is a substantial body of work examining the correlates, levels, and patterns of PA in adults and older adults, the majority of this work has been done in high-income countries [2], in which older people have been shown to have lower levels of objectively measured habitual PA than younger adults [3,4]—a phenomenon likely to exacerbate the already-high risk of cardiometabolic disease seen in older adults. Improvements in wealth and increased success in treating communicable diseases in lower- and middle-income countries (LMICs), including Sub-Saharan Africa, has led to a rapid expansion in the numbers of older people [5]. This demographic change brings new challenges, including the rise of non-communicable diseases (NCDs), driven in part by prevalent cardiometabolic risk factors such as hypertension and obesity, and by the reduction in deaths from infectious diseases, including HIV, as a result of effective antiretroviral therapies [6].

Few studies have focused on PA in older adults in LMICs, and fewer still have focused on South Africa. Reported levels of South African adults’ PA has ranged between 31.0% of adults older than 50 years being ‘physically active’ (defined in the paper as being moderately or vigorously active, based on two adapted questions in a national survey) [7] to 50% meeting PA guidelines (defined as doing >150 min/week of moderate- to vigorous-intensity PA), based on a limited sample of adults aged above 60 years in care homes [8]. One small pilot study conducted in South Africa found that the socioeconomic status, traffic, and features of the built environment were associated with objectively measured PA levels in older people [9]. Three studies have examined rural adult populations in South Africa to date, the first of which enrolled young to middle-aged adults [10], the second specified ‘community-dwelling’ older adults, but included both urban and rural adults over 50 [11], and the third included participants aged from 18 years [7]. Correlates of not meeting PA guidelines in older adults include pain, slow gait, and low grip strength [11]; while in the national survey study, physical inactivity was higher in individuals aged above 50 years, female, with at least one chronic medical condition and from rural settlements [7].

Thus, little is known about PA levels and their correlates in older South Africans from rural settings—a country with striking contrasts between urban and rural living, marked disparities in wealth and access to facilities, and a large proportion of the population still living in under-served rural areas. These contrasts between urban and rural living, including differences in the natural and built environment, family structure, amenities, type of activities necessary to live, and opportunities for recreational activity [12], may be associated with distinct patterns of PA in rural areas for older adults. Understanding the socio-demographic, functional, and health correlates of PA in older people from rural areas in South Africa is important to formulate ways to maintain PA (where it is sufficient) or increase PA (where it is low) in a time of rapid economic, demographic, and social transition. Therefore, the aims of this paper are to describe levels of self-reported PA in older rural South African men and women, and to examine associations with factors that may influence PA levels.

## 2. Materials and Methods 

We used data from the Health and Aging in Africa: A longitudinal study in an INDEPTH community (HAALSI) survey of older people. The methods for conducting this survey have been described in detail elsewhere [13]. HAALSI was conducted in 2014–2015 in the Agincourt Health and Demographic Surveillance Site (HDSS), [14] situated in the northeast of South Africa near the Mozambique border, with a population of approximately 116,000 people living in 32 villages. There is limited access to electricity or running water, and few tarred roads [14]. Ethics committee approvals for HAALSI were obtained from the University of the Witwatersrand Human Research Ethics Committee (M141159), the Harvard T.H. Chan School of Public Health Office of Human Research Administration (13-1608), and the Mpumalanga Provincial Research and Ethics Committee. The study adhered to the guidelines described in the Declaration of Helsinki Ethical Principles for Medical Research Involving Human Subjects [15]; informed consent was obtained from all participants prior to participation.

Valid PA data were available for 4538 of the 5059 individuals who consented to undergo evaluation, who formed the dataset analyzed here. The Global Physical Activity Questionnaire (GPAQ) was used to assess time spent in moderate- to vigorous-intensity physical activity (MVPA) in a usual week in the occupation, recreational, and travel domains. Participants also reported on time spent sitting or lying down on a usual day, excluding sleeping. The GPAQ has been validated for use in South African populations [16] and has previously been used with South African older adults [9]. The primary outcome variable in the logistic regression analyses was a reported MVPA level that indicated that the participant was not meeting PA guidelines (defined as reporting <150 min/week of MVPA).

Marital status was self-reported and categorized into married/cohabiting, divorced/separated, widowed, or never married. Socioeconomic status was captured using quintiles of wealth index derived from self-reported data on household assets using the method of Filmer and Pritchett [14]. Functional and cognitive capacity variables included a self-reported difficulty in walking, grip strength, and cognition. Difficulty walking was a dichotomous variable (yes/no) in response to the question in the HAALSI survey: Due to a health or memory problem do you have any difficulty with walking across a room? Grip strength was measured twice on both sides, using a Smedley Digital Hand Dynamometer (Model 12–0286, Fabrication Enterprises, Washington, DC, United States). Participants were tested in a seated position, with the tested arm held at 90 degrees of elbow flexion. The maximum reading achieved from either hand was used in the analysis, as done previously [17]. The cognition score (out of 24; a higher score indicates better cognition) was determined from a previously described cognitive test and included the following items: Orientation, word recall (both immediate and delayed), and numeracy (number series) [18,19]. This test is described in greater detail elsewhere [13].

Health variables were pain, depressive symptoms, HIV status, hypertension, diabetes mellitus, dyslipidemia, and BMI category. Experience of pain was captured using a single survey question (dichotomized yes/no): ‘Throughout our lives, most of us have had pain from time to time (such as minor headaches, sprains and toothaches). Have you had pain—other than these everyday kinds of pain—today?’ Depressive symptoms were scored based on seven of eight questions on the CES-D scale [20] and used as a continuous variable (range 0–7; a higher score indicates more depressive symptoms). One question (‘everything you did was an effort’) was excluded due to difficulties in understanding the question in the given context [17]. HIV status was based on results from dried blood spot testing as previously described [21].

For hypertension, diabetes and dyslipidemia, broad definitions were used: Diagnosis by self-report and/or having a measurement that indicated the disease, and/or the participant reported using medication to treat the disease in question at the time of testing. The measurement criteria were as follows: For hypertension, SBP ≥ 140 mmHg and/or DBP ≥ 90 mmHg; for diabetes, glucose ≥ 7 mmol/L in fasting participants or ≥11.1 mmol/L in non-fasting participants; and for dyslipidemia, elevated total cholesterol (≥6.21 mmol/L), and/or low HDL (<1.19 mmol/L), and/or elevated LDL (>4.1 mmol/L), and/or elevated triglycerides (>2.25 mmol/L)). A summary variable showing the number of these cardiometabolic risk factors was derived. Body mass index (BMI) was determined from the measured height and weight, after which participants were categorized as either underweight (<18.5 kg·m^−2^), normal weight (≥18.5 to <25 kg·m^−2^), overweight (≥25 to <30 kg·m^−2^), or obese (≥30 kg·m^−2^). 

Statistical analyses were performed using STATA (v.13, STATA Corp, College Station, TX, USA). A two-sided *p*-value of <0.05 was taken as significant for all analyses. The Shapiro-Wilk test was used to assess normality of data. Continuous data are presented as mean ± SD if normally distributed. The median (25th–75th percentile) are shown in addition to mean ± SD for data not normally distributed. To examine sex differences, Mann-Whitney-U tests (for continuous variables) and Pearson’s Chi^2^ test (for categorical variables) were completed. For MVPA (mins/week), Kruskal-Wallis tests were used to examine differences between age categories, and Pearson’s Chi^2^ test was used to compare age categories between those meeting PA guidelines and those not. Variance-weighted least-squares regression tests (VWLS) were used to determine significant trends across age groups for PA and sedentary time. Effect sizes for sex and age group differences for GPAQ variables were determined using Cohen’s *d* and the eta squared, based on the H-statistic was calculated as follows: eta2[H] = (H − k + 1)/(*n* − k).

Hierarchical multinomial logistic (mlogit) regression analyses were run to assess the factors associated with reporting a level of PA that reflected not meeting PA guidelines (the outcome variable). Model 1 included socio-demographic factors, including age, sex, wealth index, and marital status (all defined a priori). Model 2 included variables in Model 1 plus functional and cognitive capacity variables identified as likely to have a direct impact on capacity to do PA. Model 3 included all variables in Models 1 and 2 with the addition of health variables—pain, depressive symptoms, BMI category, HIV status, and ‘cluster of disease’ defined as the presence of none, 1, 2, or 3 of the following: Hypertension, diabetes mellitus, and dyslipidemia. All variables were found to be significantly correlated with meeting PA guidelines on the univariate analysis (at a significance level of *p* < 0.05) with the exception of BMI.

## 3. Results

Participant characteristics are presented in Table 1, with *p*-values indicating differences between men and women with GPAQ data. Time spent in the various domains of PA and % meeting PA guidelines, stratified by sex, are presented in Table 2. Table 3 summarizes the GPAQ results for men and women combined, by age category. 

The results of the hierarchal logistic regression are presented in Table 4. In Model 1, participants over the age of 70 years and those in the highest wealth quintile were more likely to report not meeting PA guidelines, while participants who reported being divorced/separated or married/cohabiting were less likely to report PA indicative of not meeting PA guidelines. In Model 2, participants who were male, over the age of 80 years and those in the two highest wealth quintiles were more likely to not meet PA guidelines. Additionally, participants who indicated that they experienced difficulty walking were almost four times more likely to report not meeting PA guidelines. Participants with greater grip strength and higher cognition scores were also less likely to report insufficient PA levels. In model 3, the only health variable shown to be significantly associated with insufficient PA (not meeting guidelines) was the BMI category. Obese participants were more than 1.5 times more likely to report not meeting PA guidelines. With all factors considered, the participants who were more likely to report insufficient PA were male, being over 80 years of age, in a higher wealth category (quintiles 4 or 5), to have a BMI in the obese range, and to have a poorer function in terms of walking, grip strength, and cognition.

## 4. Discussion

This study reports novel findings regarding self-reported PA and factors associated with not meeting PA guidelines (reporting insufficient PA) in a large sample of adults aged over 40 years, from rural South Africa. The majority of this sample (75.4%) were meeting PA guidelines which is consistent with another study in older adults from urban (high- and low-income) settings in South Africa, [9] where over 75% of participants exceeded the PA guideline threshold, as well as studies in younger South African populations where the proportion of participants reporting meeting PA guidelines exceeded 60% [22,23]. Similar to these studies, active transport was a major contributor to total PA. [22,23] In contrast to previous studies, the current study found that the older adults also reported high levels of recreational PA, particularly at a moderate intensity (4.6 h/week in men and 6.1 h/week in women; shown in Table 2) [11,24,25].

Irrespective of domain, older participants were less physically active and more sedentary than the younger age categories in this cohort. This is consistent with findings from other countries where both self-report and objective measures [3,4] of PA have been used to assess the relationship between age and PA. As such, it was expected to find that the oldest participants in the cohort were those most likely to report less PA, and therefore not meet the guidelines. Participants in higher wealth categories were also more likely to report insufficient PA. This is likely attributable to participants in better financial positions having more sedentary-promoting assets (such as a motor vehicle or television) at home and has been shown in a younger South African cohort of adult urban women from Soweto [23]. For functional capacity, the findings of this study confirm those reported in high-income countries, where older adults with a better function (by several definitions including cognition and ability to perform acts of daily living) were more likely to meet PA guidelines [26].

Previous studies of South African adults have shown that meeting PA guidelines is associated with better metabolic outcomes [22,23] and have indicated that this relationship is bidirectional in South African adults. It is noteworthy that in this older South African cohort, the only health variable that was shown to be associated with reporting insufficient PA was obesity. Although a bidirectional association between these measures and PA has previously been postulated in older adults (e.g., lower levels of MVPA have been associated with obesity), our results suggest that in this population, this association is not evident. However, our study was cross-sectional and longitudinal data will be required to tease out the direction of the association between PA and obesity in South African middle-aged and older adults. Cardiometabolic risk factors, denoted by diabetes, hypertension, and/or dyslipidemia, were not significantly associated with this outcome. This could be explained by the outcome variable chosen for our study (a dichotomous variable where participants report PA levels that indicate either meeting or not meeting guidelines), but may also reflect differences in how chronic diseases were reported across studies; different thresholds for disease severity or types of disease reported would be expected to have different relationships with PA levels. Diabetes in older adults is closely linked to obesity and this close relationship may explain why only one of these conditions was retained in the final model as a significant association. Hypertension is similarly associated with obesity, but as it is very prevalent in the HAALSI cohort (at 60%) it is less likely to be independently associated with PA in this cohort.

Interestingly and importantly, HIV status was not independently associated with PA. Previous work has suggested a number of HIV-specific factors, including CD4 count, opportunistic infections, and the use of antiretroviral therapy, that affect PA levels in people living with HIV [27]. This is of particular importance in this population with an HIV prevalence of 20%, many of whom are now surviving long-term due to successful treatment, but who are, as a result of medication and inflammation, exposed to an increased risk of cardiovascular events [28] and are therefore less likely to be physically active. The adverse impact of HIV on PA levels in our study population may have been attenuated by effective management, but also by survivor bias—those severely affected by HIV are more likely to have died or decline taking part in studies such as HAALSI.

It is encouraging that PA levels are high in this rural South African population, although it is possible that the international recommendations for PA are not appropriate for South African populations. Nonetheless, the increase in the age of the population and in material wealth in the future suggest that PA levels of rural South Africans may decline. Our results suggest that older people and those with higher income or greater material wealth (particularly those where active transport levels are lower) are groups at higher risk of low PA levels, and thus represent groups with whom to explore the effects of interventions to improve or maintain PA levels. A focus on physical and cognitive function is likely to be necessary if efforts to maintain or increase PA are to be successful in this population, as participants in our study with poorer cognitive scores were less likely to report meeting PA guidelines. Therefore, intervening at an early stage to prevent the ‘spiral of decline’ that characterizes the frailty syndrome (where low physical function leads to reduced PA, which then in turn leads to worsening physical performance [29]) is likely to be crucial—frailty is common in the HAALSI population, and is strongly associated with older age [30].

The strengths of this study include the large sample size that provides representation of aging adults in a South African rural area, addressing a gap in research in PA in this age group in South Africa. Another strength includes the diversity of the HAALSI data set in terms of its assessment of a wide variety of factors that can be tested for associations, which were thoroughly explored in this paper. A known limitation of using self-reported PA data includes the possibility of ‘double-reporting’, where time spent being active in one domain is duplicated in another. Additionally, self-report in an aging sample is potentially less reliable due to potential cognitive decline. However, the GPAQ is a validated tool and in the South African context has been widely used, making cross-study comparison easier. Another limitation is that this study is cross-sectional, so causality cannot be inferred. Longitudinal and intervention studies are needed to test the impact of PA levels on health and functional capacity, and future waves of data collection in HAALSI will offer the opportunity to study this. This study focused on one geographical area only, thus the results cannot necessarily be generalized to other rural South African settings, urban settings, or different countries.

## 5. Conclusions

Self-reported PA in this rural South African cohort was high, with a majority of participants meeting current recommended PA levels. However, being male, older, wealthier, and with impaired physical and cognitive performance, were associated with lower PA, suggesting targets for future interventions to maintain or improve PA levels. If the effects of low PA levels in older people in high-income countries are mirrored in LMICs, such interventions will have an important public health role in ensuring that the life years gained from the demographic transition are healthy life years, with both disease and functional impairment being minimized. Future research should therefore focus on developing interventions tailored to the needs of people in LMICs to maintain or boost PA levels.

## Figures and Tables

**Table 1:** Sample characteristics stratified by sex and participants with and without valid GPAQ data.

Chronic Disease/Health Variables	Total (*n* = 4538)	Men (*n* = 2146)	Women (*n* = 2392)	*p*-Value	Participants Missing GPAQ Data (*n* = 521)
Age—years ^a^	61.5 ± 13.0 61 (51, 71)	61.5 ± 12.7 61 (51, 71)	61.6 ± 13.4 60 (51, 71)	0.612	63.5 ± 13.1 63 (53, 73)
Age group—*n* (%)					
40–49 years	845 (18.6)	390 (18.2)	455 (19.0)	<0.001; *chi^2^(4) = 33.64*	73 (14.0)
50–59 years	1274 (28.1)	574 (26.8)	700 (29.3)		136 (26.1)
60–69 years	1159 (25.5)	591 (27.5)	568 (23.8)		145 (27.8)
70–79 years	784 (17.3)	410 (19.1)	374 (15.6)		94 (18.0)
80+ years	476 (10.5)	181 (8.4)	295 (12.3)		73 (14.0)
Marital status—*n* (%)					
Never married	261 (5.8)	150 (7.0)	111 (4.6)	<0.001; *chi^2^(3) = 686.83*	32 (6.1)
Divorced/separated	577 (12.7)	273 (12.7)	304 (12.7)		73 (14.0)
Widowed	1353 (29.8)	250 (11.7)	1103 (46.1)		188 (36.1)
Married/cohabiting	2347 (51.7)	1473 (68.6)	874 (36.5)		228 (43.8)
Wealth index—*n* (%) ^b^	*n = 4534*	*n = 2144*	*n = 2390*		
Level 1	946 (20.9)	462 (21.6)	484 (20.3)	0.681; *chi^2^(4) = 2.30*	99 (19.0)
Level 2	873 (19.3)	400 (18.7)	473 (19.8)		128 (24.6)
Level 3	866 (19.1)	405 (18.9)	461 (19.3)		122 (23.4)
Level 4	904 (19.9)	421 (19.6)	483 (20.2)		103 (19.8)
Level 5	945 (20.8)	456 (21.3)	489 (20.5)		69 (13.2)
Difficulty walking ^b^—*n* (% yes)	*n = 4530* 345 (7.6)	*n = 2141* 160 (7.5)	*n = 2389* 185 (7.7)	0.732; *chi^2^(1) = 0.12*	*n = 521* 45 (8.6)
Maximum grip strength (kg) ^a, b^	*n = 4201* 26.4 ± 9.1 25.4 (20.1, 31.8)	*n = 1985* 30.7 ± 9.3 30.6 (24.3, 36.6)	*n = 2216* 22.5 ± 6.8 22.2 (18.0, 26.5)	<0.001	*n = 490* 25.8 ± 11.5 23.5 (18.5, 29.7)
Cognition ^a, b^—total score (out of 24)	*n = 4390* 11.5 ± 4.5 12 (9, 14)	*n = 2069* 11.9 ± 4.4 12 (9, 15)	*n = 2321* 11.2 ± 4.5 11 (8, 14)	<0.001	*n = 513* 10.3 ± 4.0 11 (8, 13)
Experienced pain—*n* (% yes) ^b^	*n = 4435* 459 (10.4)	*n = 2094* 170 (8.1)	*n = 2341* 289 (12.4)	<0.001; *chi^2^(1) = 21.28*	*n = 515* 58 (11.3)
Depression symptoms ^a, b^	*n = 4421* 1.0 ± 1.4 1 (0, 1)	*n = 2086* 1.0 ± 1.3 1 (0, 1)	*n = 2335* 1.1 ± 1.5 1 (0, 1)	0.017	*n = 516* 1.4 ± 1.7 1 (0, 2)
HIV positive—*n* (% yes)	940 (20.7)	447 (20.8)	493 (20.6)	0.856; *chi^2^(1) = 0.03*	107 (20.5)
Hypertension—*n* (% yes) ^b^	*n = 4446* 2814 (63.3)	*n = 2092* 1220 (58.3)	*n = 2354* 1594 (67.7)	<0.001; *chi^2^(1) = 42.10*	*n = 507* 331 (65.3)
Diabetes mellitus (DM)—*n* (% yes) ^b^	*n = 4183* 493 (11.8)	*n = 1970* 212 (10.8)	*n = 2213* 281 (12.7)	0.053; *chi^2^(1) = 3.76*	*n = 469* 66 (14.1)
Dyslipidemia—*n* (% yes) ^b^	*n = 3818* 1664 (43.6)	*n = 1760* 784 (44.6)	*n = 2058* 880 (42.8)	0.267; *chi^2^(1) = 1.23*	*n = 433* 198 (45.7)
Cluster of disease (hypertension + DM + dyslipidemia)—*n* (% yes) ^b^	*n = 4201*	*n = 1957*	*n = 2244*		*n = 479*
None	1011 (24.1)	534 (27.3)	477 (21.3)	<0.001; *chi^2^(3) = 23.33*	107 (22.3)
1	1886 (44.9)	861 (44.0)	1025 (45.7)		212 (44.3)
2	1103 (26.3)	480 (24.5)	623 (27.8)		127 (26.5)
3	201 (4.8)	82 (4.2)	119 (5.3)		33 (6.9)
BMI category—*n* (%) ^b^	*n = 4191*	*n = 1973*	*n = 2218*	<0.001; *chi^2^(3) = 396.87*	*n = 492*
Normal weight	1539 (36.7)	928 (47.0)	611 (27.6)		179 (36.4)
Underweight	227 (5.4)	166 (8.4)	61 (2.8)		27 (5.5)
Overweight	1193 (28.5)	563 (28.5)	630 (28.4)		133 (27.0)
Obese	1232 (29.4)	316 (16.0)	916 (41.3)		153 (31.1)

^a^ Continuous variables presented as mean ± SD and median (25th–75th percentile); ^b^ where the number of participants differs from the total cohort; the n is shown in italics; Mann-Whitney-U tests used to determine differences between men and women for continuous variables; chi^2^ test used for categorical variables (chi^2^ values are given in *italics*). *p*-values indicate statistical significance for differences between men and women.

**Table 2:** Global physical activity questionnaire (GPAQ) results for participants with valid GPAQ data, stratified by sex.

GPAQ Variable	Total (*n* = 4538)	Men (*n* = 2146)	Women (*n* = 2392)	*p*-Value	Cohen’s d
Occupational VPA (min·wk^−1^)	7.3 ± 125.1 0 (0, 0)	9.1 ± 142.4 0 (0, 0)	5.6 ± 107.2 0 (0, 0)	0.796	0.03
Occupational MPA (min·wk^−1^)	9.9 ± 147.2 0 (0, 0)	9.3 ± 156.9 0 (0, 0)	10.4 ± 137.9 0 (0, 0)	0.338	−0.01
Occupational MVPA (min·wk^−1^)	17.1 ± 203.7 0 (0, 0)	18.4 ± 219.8 0 (0, 0)	15.9 ± 188.1 0 (0, 0)	0.690	0.01
Transport MPA (min·wk^−1^)	430.2 ± 493.6 270.0 (60.0, 600.0)	494.7 ± 545.8 360.0 (90.0, 720.0)	372.3 ± 433.6 240.0 (60.0, 480.0)	<0.001	0.25
Recreational VPA (min·wk^−1^)	146.8 ± 374.3 0 (0, 0)	154.2± 383.8 0 (0, 30.0)	140.4 ± 365.6 0 (0, 0)	0.014	0.04
Recreational MPA (min·wk^−1^)	322.8 ± 586.2 0 (0, 360.0)	278.0 ± 531.1 0 (0, 360.0)	363.0 ± 628.9 0 (0, 480.0)	0.003	−0.15
Recreational MVPA (min·wk^−1^)	469.6 ± 816.7 0 (0, 630.0)	432.2 ± 778.6 0 (0, 600.0)	503.2 ± 848.1 0 (0, 765.0)	0.051	−0.09
Total MVPA (min·wk^−1^)	916.9 ± 1061.4 549.5 (150.0, 1260.0)	945.3 ± 1083.5 600.0 (160.0, 1260.0)	891.4 ± 1040.7 480.0 (150.0, 1260.0)	0.061	0.05
Meeting PA guidelines (n, %)	3421, 75.4%	1622, 75.6%	1799, 75.2%	0.771; *chi^2^(1) = 0.09*	
Sitting time—weekdays (h.d^−1^)	3.5 ± 2.3 3.0 (2.0, 5.0)	3.6 ± 2.4 3.0 (2.0, 5.0)	3.4 ± 2.2 3.0 (2.0, 5.0)	0.004	0.09
Sitting time—weekend days (h.d^−1^)	3.5 ± 2.3 3.0 (2.0, 5.0)	3.6 ± 2.4 3.0 (2.0, 5.0)	3.4 ± 2.3 3.0 (2.0, 5.0)	<0.001	0.09

Data presented as mean ± SD and median (25th–75th percentile), GPAQ: Global Physical Activity Questionnaire; MPA: Moderate-intensity Physical Activity; d: day; wk: week; MVPA: Moderate- to Vigorous-intensity Physical Activity; VPA: Vigorous Physical Activity; SB: Sedentary Behavior; Mann-Whitney-U tests used to determine differences between men and women for continuous variables; Cohen’s d used to determine effect size differences between men and women; chi^2^ test used for categorical variables (chi^2^ values are given in *italics*).

**Table 3:** Global physical activity questionnaire (GPAQ) results stratified by age category.

GPAQ Variable	40–49 y (*n* = 845)	50–59 y (*n* = 1274)	60–69 y (*n* = 1159)	70–79 y (*n* = 784)	80+ y (*n* = 476)	*p*-Value	Eta Squared	*p*-Value for Trend
Occupational VPA (min·wk^−1^)	7.5 ± 128.3 0 (0, 0)	17.0 ± 188.7 0 (0, 0)	4.3 ± 99.8 0 (0, 0)	0 ± 0 0 (0, 0)	0 ± 0 0 (0, 0)	<0.001	0.003	0.348; *chi^2^(1) = 0.88*
Occupational MPA (min·wk^−1^)	19.0 ± 221.2 0 (0, 0)	16.2 ± 174.6 0 (0, 0)	4.6 ± 96.3 0 (0, 0)	3.4 ± 96.4 0 (0, 0)	0 ± 0 0 (0, 0)	0.004	0.003	0.013; *chi^2^(1) = 6.20*
Occupational MVPA (min·wk^−1^)	26.5 ± 255.6 0 (0, 0)	33.2 ± 279.7 0 (0, 0)	8.9 ± 148.2 0 (0, 0)	3.4 ± 96.4 0 (0, 0)	0 ± 0 0 (0, 0)	<0.001	0.005	<0.001; *chi^2^(1) = 12.89*
Transport MPA (min·wk^−1^)	501.0 ± 547.7 300.0 (120.0, 720.0)	479.0 ± 474.3 360.0 (120.0, 720.0)	465.8 ± 524.7 300.0 (90.0, 630.0)	360.3 ± 442.6 210.0 (60.0, 480.0)	202.4 ± 344.7 60.0 (0, 240.0)	<0.001	0.055	<0.001; *chi^2^(1) = 206.25*
Recreational VPA (min·wk^−1^)	204.56 ± 450.7 0 (0, 240.0)	162.3 ± 384.6 0 (0, 20.0)	144.8 ± 370.8 0 (0, 0)	108.2 ± 311.0 0 (0, 0)	71.2 ± 267.8 0 (0, 0)	<0.001	0.013	<0.001; *chi^2^(1) = 59.20*
Recreational MPA (min·wk^−1^)	394.1 ± 654.1 0 (0, 540.0)	368.6 ± 633.8 0 (0, 480.0)	360.5 ± 606.6 0 (0, 540.0)	238.3 ± 464.9 0 (0, 240.0)	121.1 ± 347.9 0 (0, 30.0)	<0.001	0.023	<0.001; *chi^2^(1) = 155.00*
Recreational MVPA (min·wk^−1^)	598.8 ± 924.8 150.0 (0, 840.0)	530.9 ± 875.2 0 (0, 840.0)	505.3 ± 835.9 0 (0, 720.0)	346.4 ± 646.4 0 (0, 420.0)	192.3 ± 510.8 0 (0, 120.0)	<0.001	0.026	<0.001; *chi^2^(1) = 155.67*
Total MVPA (min·wk^−1^)	1126.2 ± 1163.0 780.0 (270.0, 1500.0)	1043.0 ± 1096.1 720.0 (240.0, 1440.0)	979.9 ± 1102.4 600.0 (180.0, 1380.0)	710.2 ± 861.5 375.0 (90.0, 1020.0)	394.8 ± 690.3 120.0 (0, 430.0)	<0.001	0.069	<0.001; *chi^2^(1) = 297.99*
Meeting PA guidelines (n, %)	695, 82.3%	1038, 81.5%	910, 78.5%	549, 70.0%	229, 48.1%	<0.001; *chi^2^(4) = 256.03*		<0.001; *chi^2^(1) = 149.61*
Weekday Sitting (h·d^−1^)	3.5 ± 2.1 3.0 (2.0, 5.0)	3.4 ± 2.1 3.0 (2.0, 5.0)	3.6 ± 2.2 3.0 (2.0, 5.0)	3.7 ± 2.4 3.0 (2.0, 5.0)	3.7 ± 2.9 3.0 (1.0, 6.0)	0.275	<0.001	0.009; *chi^2^(1) = 6.74*
Weekend day Sitting (h·d^−1^)	3.5 ± 2.1 3.0 (2.0, 5.0)	3.4 ± 2.2 3.0 (2.0, 4.5)	3.6 ± 2.3 3.0 (2.0, 5.0)	3.6 ± 2.5 3.0 (2.0, 5.0)	3.6 ± 2.9 3.0 (1.0, 5.8)	0.267	<0.001	0.122; *chi^2^(1) = 2.39*

Data presented as mean ± SD and median (25th–75th percentile); GPAQ: Global Physical Activity Questionnaire; MPA: Moderate-intensity Physical Activity; MVPA: Moderate- to Vigorous-intensity Physical Activity; VPA: Vigorous Physical Activity; d: day; wk: week; y: year; SB: Sedentary Behavior; Kruskal-Wallis tests used to determine differences between age groups; eta squared, based on the H-statistic, used to determine effect size differences between age groups; chi^2^ test used for categorical variables (chi^2^ values are given in *italics*). Variance-weighted least-squares regression test used to determine the *p*-value for trend (in increasing age).

**Table 4:** Hierarchical regression model showing variables associated with not meeting guidelines for self-reported physical activity according to the GPAQ.

Outcome Variable: Not Meeting PA Guidelines	Model 1: Socio-Demographic (*n* = 4534)			Model 2: Socio-Demographic + Functional and Cognitive Capacity (*n* = 4116)			Model 3: Socio-Demographic + Functional and Cognitive Capacity + Chronic Disease/Health (*n* = 3729)		
	RRR	95% CI	*p*-Value	RRR	95% CI	*p*-Value	RRR	95% CI	*p*-Value
Age									
40–49 y (ref)	1	-	-	1	-	-	1	-	-
50–59 y	1.07	0.85−1.34	0.588	0.84	0.65−1.09	0.198	0.84	0.63−1.12	0.224
60–69 y	1.25	0.99−1.58	0.057	0.87	0.66−1.14	0.310	0.87	0.64−1.17	0.347
70–79 y	1.90	1.49−2.44	<0.001	0.96	0.71−1.30	0.793	0.95	0.68−1.33	0.763
80 + y	4.64	3.54−6.09	<0.001	1.70	1.21−2.40	0.002	1.73	1.18−2.54	0.005
Sex									
Female (ref)	1	-	-	1	-	-	1	-	-
Male	1.14	0.97−1.33	0.106	1.23	1.02−1.50	0.034	1.40	1.11−1.75	0.004
Wealth index									
Level 1 (lowest-ref)	1	-	-	1	-	-	1	-	-
Level 2	1.17	0.94−1.46	0.164	1.26	0.98−1.63	0.071	1.31	0.99−1.74	0.055
Level 3	0.99	0.79−1.25	0.969	1.12	0.86−1.46	0.400	1.13	0.85−1.50	0.407
Level 4	1.11	0.89−1.39	0.345	1.45	1.12−1.87	0.005	1.43	1.08−1.88	0.013
Level 5 (highest)	1.29	1.04−1.61	0.022	1.79	1.38−2.32	<0.001	1.70	1.29−2.27	<0.001
Marital status									
Never married (ref)	1	-	-	1	-	-	1	-	-
Divorced/separated	0.64	0.44−0.91	0.014	1.01	0.64−1.61	0.961	0.98	0.57−1.68	0.941
Widowed	0.93	0.66−1.29	0.653	1.31	0.84−2.03	0.228	1.32	0.79−2.19	0.286
Married/cohabiting	0.69	0.50−0.94	0.019	1.25	0.82−1.88	0.299	1.27	0.78−2.06	0.333
	LR chi^2^(12) = 252.97, *p* < 0.000, Pseudo R^2^ = 0.050								
Difficulty walking									
No (ref)				1	-	-	1	-	-
Yes				3.93	2.95−5.23	<0.001	3.42	2.48−4.71	<0.001
Max grip strength									
Score, continuous (range 3.2–93.1 kg)				0.99	0.97–1.00	0.014	0.98	0.97−1.00	0.008
Cognition									
Score, continuous (range 0–24)				0.89	0.87−0.91	<0.001	0.90	0.88−0.92	<0.001
				LR chi^2^(15) = 411.91, *p* < 0.000, Pseudo R^2^ = 0.097					
Pain									
No (ref)							1	-	-
Yes							0.81	0.62−1.05	0.116
Depressive symptoms									
Score, continuous (range 0–7)							1.06	1.00−1.13	0.056
BMI category									
Normal weight (ref)							1	-	-
Underweight							1.08	0.72−1.61	0.712
Overweight							1.19	0.95−1.50	0.124
Obese							1.61	1.26−2.04	<0.001
HIV status									
Negative (ref)							1	-	-
Positive							0.75	0.77−1.23	0.832
Cluster of disease									
None (ref)							1	-	-
Yes—1							0.92	0.74−1.16	0.494
Yes—2							1.11	0.87−1.43	0.397
Yes—3							1.03	0.68−1.57	0.882
							LR chi^2^(24) = 332.28, *p* < 0.000, Pseudo R^2^ = 0.090		

## References

[1] Wahid A., Manek N., Nichols M., Kelly P., Foster C., Webster P., Kaur A., Smith C.F., Wilkins E., Rayner M. (2016). Quantifying the Association Between Physical Activity and Cardiovascular Disease and Diabetes: A Systematic Review and Meta-Analysis. J. Am. Heart Assoc..

[2] Bauman A.E., Reis R.S., Sallis J.F., Wells J.C., Loos R.J.F., Martin B.W. (2012). Correlates of physical activity: Why are some people physically active and others not?. Lancet.

[3] Jefferis B.J., Sartini C., Lee I.-M., Choi M., Amuzu A., Gutierrez C., Casas J.P., Ash S., Lennnon L.T., Wannamethee S.G. (2014). Adherence to physical activity guidelines in older adults, using objectively measured physical activity in a population-based study. BMC Public Health.

[4] Colley R.C., Garriguet D., Janssen I., Craig C.L., Clarke J., Tremblay M. (2011). Physical activity of Canadian adults: Accelerometer results from the 2007 to 2009 Canadian Health Measures Survey. Health Rep..

[5] Bor J., Herbst A.J., Newell M.-L., Bärnighausen T. (2013). Increases in Adult Life Expectancy in Rural South Africa: Valuing the Scale-Up of HIV Treatment. Science.

[6] GBD 2015 Mortality and Causes of Death Collaborators (2016). Global, regional, and national life expectancy, all-cause mortality, and cause-specific mortality for 249 causes of death, 1980–2015: A systematic analysis for the Global Burden of Disease Study 2015. Lancet.

[7] Mlangeni L., Makola L., Naidoo I., Chibi B., Sokhela Z., Silimfe Z., Mabaso M. (2018). Factors Associated with Physical Activity in South Africa: Evidence from a National Population Based Survey. Open Public Health J..

[8] Aro A.A., Agbo S., Omole O.B. (2018). Factors influencing regular physical exercise among the elderly in residential care facilities in a South African health district. Afr. J. Prim. Health Care Fam. Med..

[9] Kolbe-Alexander T., Pacheco K., Tomaz S.A., Karpul D., Lambert E.V. (2015). The relationship between the built environment and habitual levels of physical activity in South African older adults: A pilot study. BMC Public Health.

[10] Cook I., Alberts M., Brits J.S., Choma S., Mkhonto S.S. (2010). Descriptive Epidemiology of Ambulatory Activity in Rural, Black South Africans. Med. Sci. Sports Exerc..

[11] Koyanagi A., Stubbs B., Smith L., Gardner B., Vancampfort D. (2017). Correlates of physical activity among community-dwelling adults aged 50 or over in six low- and middle-income countries. PLoS ONE.

[12] Kärmeniemi M., Lankila T., Ikäheimo T.M., Koivumaa-Honkanen H., Korpelainen R. (2018). The Built Environment as a Determinant of Physical Activity: A Systematic Review of Longitudinal Studies and Natural Experiments. Ann. Behav. Med..

[13] Gomez-Olive F.X., Montana L., Wagner R.G., Kabudula C.W., Rohr J.K., Kahn K., Bärnighausen T., Collinson M., Canning D., Gaziano T. (2018). Cohort Profile: Health and Ageing in Africa: A Longitudinal Study of an INDEPTH Community in South Africa (HAALSI). Int. J. Epidemiol..

[14] Kahn K., Collinson M.A., Gomez-Olive F.X., Mokoena O., Twine R., Mee P., Afolabi S.A., Clark B.D., Kabudula C.W., Khosa A. (2012). Profile: Agincourt Health and Socio-demographic Surveillance System. Int. J. Epidemiol..

[15] World Medical Association (2013). World Medical Association Declaration of Helsinki. JAMA.

[16] Bull F.C., Maslin T.S., Armstrong T. (2009). Global Physical Activity Questionnaire (GPAQ): Nine Country Reliability and Validity Study. J. Phys. Act. Health.

[17] Payne C.F., Gómez-Olivé F.X., Kahn K., Berkman L. (2017). Physical Function in an Aging Population in Rural South Africa: Findings from HAALSI and Cross-National Comparisons with HRS Sister Studies. J. Gerontol. Series B Psychol. Sci. Soc. Sci..

[18] Sonnega A., Faul J.D., Ofstedal M.B., Langa K.M., Phillips J.W.R., Weir D.R. (2014). Cohort Profile: The Health and Retirement Study (HRS). Int. J. Epidemiol..

[19] Humphreys G.W., Duta M., Montana L., Demeyere N., McCrory C., Rohr J., Kahn K., Tollman S., Berkman L. (2016). Cognitive Function in Low-Income and Low-Literacy Settings: Validation of the Tablet-Based Oxford Cognitive Screen in the Health and Aging in Africa: A Longitudinal Study of an INDEPTH Community in South Africa (HAALSI). J. Gerontol. Ser. B Psychol. Sci. Soc. Sci..

[20] Radloff L.S. (1977). The CES-D Scale: A Self-Report Depression Scale for Research in the General Population. Appl. Psychol. Meas..

[21] Rohr J.K., Gómez-Olivé F.X., Rosenberg M., Manne-Goehler J., Geldsetzer P., Wagner R.G., Houle B., Salomon J.A., Kahn K., Tollman S. (2017). Performance of self-reported HIV status in determining true HIV status among older adults in rural South Africa: A validation study. J. Int. AIDS Soc..

[22] Dickie K., Micklesfield L., Chantler S., Lambert E.V., Goedecke J.H. (2014). Meeting physical activity guidelines is associated with reduced risk for cardiovascular disease in black South African women; a 5.5-year follow-up study. BMC Public Health.

[23] Gradidge P.J., Crowther N.J., Chirwa E.D., Norris S.A., Micklesfield L. (2014). Patterns, levels and correlates of self-reported physical activity in urban black Soweto women. BMC Public Health.

[24] Balis L.E., Sowatey G., Ansong-Gyimah K., Ofori E., Harden S.M. (2019). Older Ghanaian adults’ perceptions of physical activity: An exploratory, mixed methods study. BMC Geriatr..

[25] Assah F., Mbanya J.C., Ekelund U., Wareham N., Brage S. (2015). Patterns and correlates of objectively measured free-living physical activity in adults in rural and urban Cameroon. J. Epidemiol. Community Health.

[26] Shah R.C., Buchman A.S., Leurgans S.E., Boyle P.A., Bennett D.A. (2012). Association of total daily physical activity with disability in community-dwelling older persons: A prospective cohort study. BMC Geriatr..

[27] Vancampfort D., Mugisha J., De Hert M., Probst M., Firth J., Gorczynski P., Stubbs B. (2016). Global physical activity levels among people living with HIV: A systematic review and meta-analysis. Disabil. Rehabil..

[28] Samaras K. (2012). The Burden of Diabetes and Hyperlipidemia in Treated HIV Infection and Approaches for Cardiometabolic Care. Curr. HIV/AIDS Rep..

[29] Fried L.P., Tangen C.M., Walston J., Newman A.B., Hirsch C., Gottdiener J., Seeman T., Tracy R., Kop W.J., Burke G. (2001). Frailty in older adults: Evidence for a phenotype. J. Gerontol. A Boil. Sci. Med. Sci..

[30] Payne C.F., Wade A.N., Kabudula C.W., Davies J., Chang A.Y., Gomez-Olive F.X., Kahn K., Berkman L.F., Tollman S., Salomon J.A. (2017). Prevalence and correlates of frailty in an older rural African population: Findings from the HAALSI cohort study. BMC Geriatr..

